# Enhanced Cationic Charge is a Key Factor in Promoting Staphylocidal Activity of α-Melanocyte Stimulating Hormone via Selective Lipid Affinity

**DOI:** 10.1038/srep31492

**Published:** 2016-08-16

**Authors:** Jyotsna Singh, Seema Joshi, Sana Mumtaz, Nancy Maurya, Ilora Ghosh, Shivangi Khanna, Vivek T. Natarajan, Kasturi Mukhopadhyay

**Affiliations:** 1 Antimicrobial Research Laboratory, School of Environmental Sciences, Jawaharlal Nehru University, New Delhi-110067, India; 2 Biochemistry and Environmental Toxicology Laboratory, School of Environmental Sciences, Jawaharlal Nehru University, New Delhi-110067, India; 3 CSIR-Institute of Genomics and Integrative Biology, Mathura Road, New Delhi-110020, India

## Abstract

The steady rise in antimicrobial resistance poses a severe threat to global public health by hindering treatment of an escalating spectrum of infections. We have previously established the potent activity of α-MSH, a 13 residue antimicrobial peptide, against the opportunistic pathogen *Staphylococcus aureus*. Here, we sought to determine whether an increase in cationic charge in α-MSH could contribute towards improving its staphylocidal potential by increasing its interaction with anionic bacterial membranes. For this we designed novel α-MSH analogues by replacing polar uncharged residues with lysine and alanine. Similar to α-MSH, the designed peptides preserved turn/random coil conformation in artificial bacterial mimic 1,2-dimyristoyl-sn-glycero-3-phosphocholine:1,2-dimyristoyl-sn-glycero-3-phospho-rac-(1-glycerol) (7:3, w/w) vesicles and showed preferential insertion in the hydrophobic core of anionic membranes. Increased cationic charge resulted in considerable augmentation of antibacterial potency against MSSA and MRSA. With ~18-fold better binding than α-MSH to bacterial mimic vesicles, the most charged peptide KKK-MSH showed enhanced membrane permeabilization and depolarization activity against intact *S. aureus*. Scanning electron microscopy confirmed a membrane disruptive mode of action for KKK-MSH. Overall, increasing the cationic charge improved the staphylocidal activity of α-MSH without compromising its cell selectivity. The present study would help in designing more effective α-MSH-based peptides to combat clinically relevant staphylococcal infections.

The steady rise in community acquired and nosocomial infections due to *S. aureus*, especially methicillin-resistant *S. aureus* (MRSA), reported over the decades, reflects an expanding reservoir of patient populations at risk for such infections^1^. MRSA is the major causative agent for skin and soft tissue infections, endocarditis, osteomyelitis, necrotizing pneumonia and septicaemia with associated mortality rates up to 20% in severe cases of infection. Of special concern is the ability of MRSA to withstand multiple classes of antibiotics such as β-lactams, macrolides, quinolones, tetracycline and last resort drugs like vancomycin. Emerging resistance trends of *S. aureus*, along with its innate virulence mechanism, demands a desperate need to identify new antimicrobial agents with novel modes of action^2^. Cationic antimicrobial peptides (CAMPs) with direct bactericidal, membrane disruptive mode of action and low target-based resistance development are potential candidates for such a new class of antibiotics^3^,^4^. CAMPs are small (12–50 amino acids) molecules involved in innate immunity as the first line of defence in almost all life forms from bacteria to humans. With a balance between positive charge (+2 to +9) and hydrophobic residues, CAMPs adopt amphipathic structures in the vicinity of biological membranes which allows direct killing of microbes by disruption of anionic bacterial membranes^5^,^6^. The capability of CAMPs to synergize with known antibiotics along with their immune-modulatory abilities can be advantageous for their development as potential leads over conventional antibiotics and serve greatly towards making them therapeutically viable^5^.

Alpha-Melanocyte stimulating hormone (α-MSH) is an endogenous neuropeptide which apart from its melanogenic properties, is known for its antipyretic and anti-inflammatory effects^7^,^8^. A linear tridecapeptide (Ac-Ser^1^-Tyr^2^-Ser^3^-Met^4^-Glu^5^-His^6^-Phe^7^-Arg^8^-Trp^9^-Gly^10^-Lys^11^-Pro^12^-Val^13^-NH_2_), α-MSH results from the cleavage of its precursor polypeptide pro-opiomelanocortin (POMC). The presence and distribution of this neuropeptide in non-pigmentary barrier and immune cells, namely neutrophils, monocytes, fibroblasts and keratinocytes, further signified its role in immune-modulatory functions in the host^9^,^10^. Notably, *in vivo* anti-inflammatory as well as other beneficial effects of α-MSH and related peptides in animal models (for e.g. in asthma, obesity, inflammatory bowel disease, rheumatoid arthritis) have also been validated by various research groups^11^,^12^,^13^. At molecular level, through interactions with various melanocortin receptors (MC1R, MC3R, MC4R and MC5R) present on the cell surface, α-MSH interferes with cyclic adenosine monophosphate (cyclic-AMP) signaling leading to the downregulation of pro-inflammatory mediators such as nuclear factor *κβ* (NF-*κβ*), thus protecting the brain and peripheral organs from inflammatory conditions^14^,^15^. Additionally, α-MSH has been shown to mediate its anti-inflammatory effects through signaling pathways such as calcium flux and Jak/STAT activation^16^,^17^.

The prominent sequence and structural similarity of this anti-inflammatory peptide with CAMPs was implicated towards its role in host defense^18^. Cutuli *et al*. first established direct *in vitro* antimicrobial effect of α-MSH and its carboxy-terminal tripeptide α-MSH(11–13) against *Candida albicans* and *S. aureus* which opened a promising avenue to exploit these peptides as antimicrobial agents^19^. Further, based on structure-activity relationships, more potent α-MSH(6–13)-based peptidomimetics with candidacidal and antibacterial activity have been reported^20^,^21^. Over the past decade, our group has established rapid antibacterial activity of α-MSH against *S. aureus* under physiological high salt, serum and various pH conditions^22^. We also demonstrated that C-terminal fragments of α-MSH, including α-MSH(6–13) and tripeptide α-MSH(11–13), possessed equivalent antimicrobial activities compared to full length peptide whereas the N-terminal fragment, α-MSH(1–5), did not show any activity^23^. In the same study, we also demonstrated that α-MSH and its active fragments cause permeabilization and depolarization of *S. aureus* membrane. Encouragingly, α-MSH showed activity against multiple clinical isolates (75) of *S. aureus* while hampering DNA replication and protein synthesis without compromising cell selectivity^24^,^25^.

Recently, we also showed that α-MSH preferentially interacts with negatively charged bacterial mimic membranes viz., DMPG (1,2-dimyristoyl-sn-glycero-3-phospho-rac-(1-glycerol) sodium salt) containing vesicles, as compared to neutral mammalian mimic membranes while retaining its secondary structure^26^. α-MSH also led to membrane permeabilization of vesicles leading to leakage of the vesicular contents in a concentration as well as vesicular composition-dependent manner. Several studies have established that the presence of high levels of cationic residues in CAMPs may result in a stronger preference for interactions with anionic bacterial membranes^27^,^28^. These concepts are further supported by recent observations that net increase in cationic surface charge of *S. aureus* membrane confers reduced susceptibility to CAMPs^29^. Enhanced efficacy of CAMPs through an increase in cationic charge without an increase in toxicity to the host cells has been achieved in several studies^30^,^31^. Therefore, in this study, in order to improve the efficacy of α-MSH, we designed four novel analogues using cationic charge as the physicochemical parameter. For the designed analogues, secondary structures induced upon incorporation of cationic amino acids, as well as interaction with biological membranes were evaluated using artificial bacterial mimic membranes. Further, the work was extended to both live bacterial cells and mammalian cells to determine their antibacterial and cytotoxic activities. Additionally, we also quantified levels of intracellular cyclic-AMP elicited upon peptide treatment in mouse melanocytes. Finally, we evaluated how increasing cationic charge may influence bacterial membrane disruption through more efficient binding to anionic lipid vesicles.

## Results

### Design of novel cationic analogues of α-MSH

Towards improving the staphylocidal efficacy of α-MSH, in our present study, we altered its primary sequence by incorporating Lys residues to increase the overall cationic charge. Having previously established that the C-terminal residues 6–13 in α-MSH are indispensable for its antimicrobial activity with α-MSH(6–13) and α-MSH(11–13) being active as standalone molecules^23^, we focused on N-terminal residues 1–5, replacing them with Lys residues, towards the generation of novel α-MSH analogues. The polar residues at N-terminal of the sequence were systematically replaced, thereby increasing the cationic charge resulting in K-MSH (Ser^**1**^ replaced with Lys^**1**^, net charge +2) and KK-MSH (Ser^**1,3**^ replaced with Lys^**1,3**^, net charge +3) (Table 1). The negatively charged Glu residue at the 5^th^ position was replaced with Ala in KKA-MSH, leading to enhancement of the overall cationic charge (+4).

The Ala in KKA-MSH was further replaced with Lys at 5^th^ position, resulting in peptide KKK-MSH with a net cationic charge of +5. All analogues were custom synthesized with N-terminal acetylation and C-terminal amidation. Table 1 summarizes the name, sequences, charge and % hydrophobicity based on RP-HPLC elution of α-MSH and the designed peptides. As shown in Table 1, upon incorporation of Lys or Ala residues, the cationic charge progressively increased from +1 to +5, however, the relative decrease in % hydrophobicity was not considerable (~4%) among the analogues as compared to α-MSH.

### Designed peptides showed similar secondary structure as α-MSH in different membrane-mimetic environments

**Figure 1:**
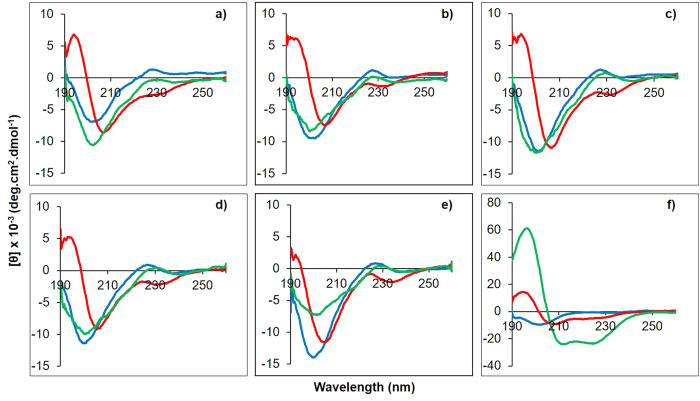
CD spectra of α-MSH and designed analogues. (**a**) α-MSH, (**b**) K-MSH, (**c**) KK-MSH, (**d**) KKA-MSH, (**e**) KKK-MSH and (**f**) Magainin II in the presence of buffer (blue), 50% TFE (v/v) (red) and DMPC:DMPG (7:3, w/w) SUVs (green). Peptide and lipid concentrations were 50 μM and 726 μM, respectively (L:P = 14.5:1). The mean residue ellipticity [θ] was plotted against wavelength. Each plot represents average of three scans.

To elucidate the secondary structure, circular dichroism (CD) spectra of all peptides, including an α-helix forming peptide magainin II as standard, were acquired in different environments viz., 5 mM sodium phosphate buffer, 50% TFE (v/v) and bacterial mimic DMPC:DMPG SUVs (7:3 and 1:1, w/w). Figure 1a–f shows the representative CD spectra expressed as deg.cm^2^.dmol^−1^ vs. wavelength. As reported previously, in buffer α-MSH exhibited random coil conformation^26^,^32^ while all the designed analogues also showed random coil conformation with a minimum in the range of 200–204 nm. All the peptides showed a propensity towards helical conformation in helix inducing solvent TFE (50% v/v) with maxima around 192–194 nm and double minima around 204–207 nm and 219–224 nm. However, in bacterial mimic DMPC:DMPG vesicles at a lipid to peptide ratio of 14.5:1, there were no appreciable changes in the secondary structure of either α-MSH or studied analogues as compared to spectrum in buffer^26^,^32^ (Fig. 1). Additional CD data of α-MSH and designed analogues in different composition of DMPC:DMPG SUVs (7:3 and 1:1, w/w) at a L:P ratio of 100:1 was also acquired (see Supplementary Fig. S1). The high L:P ratio was studied to ensure complete binding of peptides to lipids so that there were no free peptides during CD measurements. The results showed no major change upon increasing the L:P ratio from 14.5:1 to 100:1. Almost identical CD data was obtained for all peptides in 7:3 (w/w) and 1:1 (w/w) lipid compositions except for K-MSH and KK-MSH which showed slightly less ordered conformation in 1:1 (w/w) DMPC:DMPG SUVs. De convolution of CD data (CD Pro software) indicated a mixed, predominantly random coil with β-turn conformation for α-MSH and all the designed peptides in DMPC:DMPG (7:3, w/w) vesicles. The standard peptide magainin II showed random coil conformation in buffer (Fig. 1f) and switched to a characteristic α-helical conformation in bacterial mimic SUVs.

### The designed peptides and α-MSH showed preferential interaction with artificial bacterial mimic membranes

**Figure 2:**
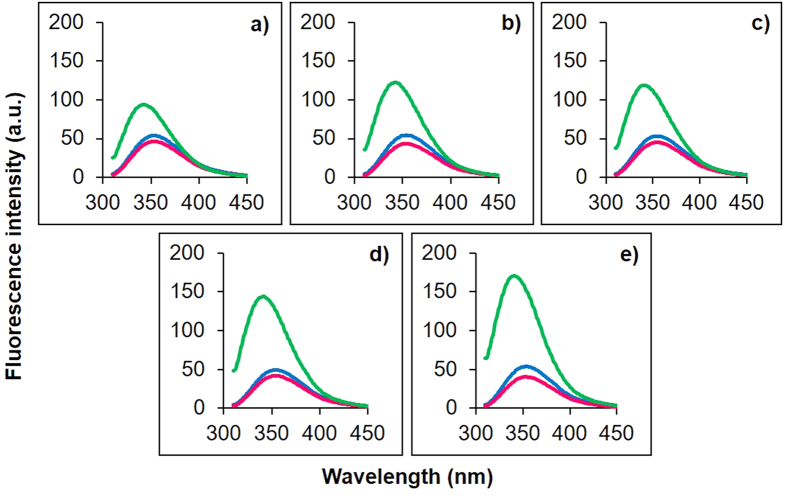
Tryptophan fluorescence emission maxima of α-MSH and designed analogues in different environments. (**a**) α-MSH, (**b**) K-MSH, (**c**) KK-MSH, (**d**) KKA-MSH and (**e**) KKK-MSH were added to buffer (blue), DMPC:DMPG (7:3, w/w) SUVs (green) and DMPC SUVs (pink). Peptide and lipid concentrations were 12.5 μM and 726 μM, respectively. Values from three scans were averaged per sample.

We further evaluated insertion depth of designed peptides in artificial bacterial and mammalian mimic membranes using Trp fluorescence. In general, a blue shift in emission maxima along with an increase in quantum yield is expected when a peptide interacts with the vesicles due to alteration in the Trp microenvironments^33^. Since all the peptides contain one Trp residue at 9^th^ position, we used it as a probe to monitor changes in the local environment of the Trp residue. We measured Trp fluorescence in four different environments including 10 mM TES buffer, DMPC SUVs, DMPC:DMPG (7:3, w/w) SUVs and DMPC:DMPG (1:1, w/w) SUVs. Similar to our previous report on α-MSH, in buffer the Trp residue, in all the peptides, showed emission maximum around 350 nm which is characteristic of Trp residue in α-MSH in polar aqueous environments^26^ (Fig. 2).

Compared to buffer, only minor increase in emission intensity and blue shift (0–3 nm) in the position of emission maximum were observed for all the peptides in neutral DMPC SUVs (Fig. 2 and Table 2). In contrast, in bacterial mimic DMPC:DMPG (7:3 and 1:1, w/w) SUVs, appreciable blue shifts (10–14 nm) were observed in the position of emission maxima of Trp residue along with ~3-fold increase in emission intensity (Fig. 2) for all studied peptides. This clearly indicates that unlike in neutral lipid vesicles, the Trp residue of all designed analogues was partitioning in the hydrophobic core of negatively charged lipid vesicles.

### Novel analogues showed enhanced potency against *S. aureus*

**Figure 3:**
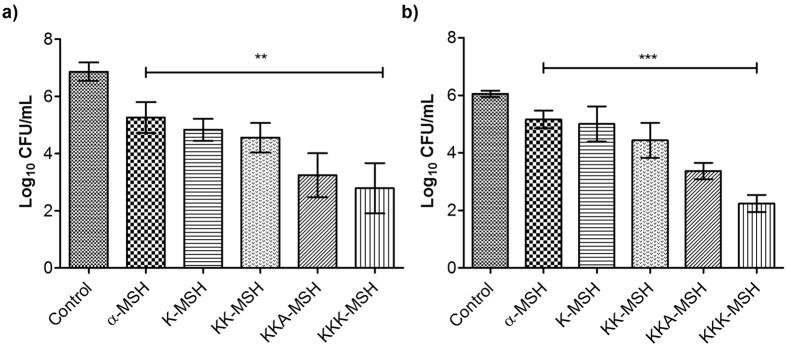
Antibacterial activity of α-MSH and designed analogues against *S. aureus.* Effect of 10 μM α-MSH and its analogues on viability of (**a**) 10^7^ CFU/mL MSSA ATCC 29213 and (**b**) 10^6^ CFU/mL MRSA ATCC 33591. Each data point is an average of three independent experiments and error bar represents S.D.

Antibacterial activity of designed analogues was compared to that of α-MSH by performing 2 h killing assay against logarithmic phase of MSSA (ATCC 29213) and MRSA (ATCC 33591) and the results are presented in Fig. 3. In the cases of both MSSA and MRSA, the increase in cationicity of the designed peptides resulted in significant killing leading to a reduction in Log_10_ CFU/mL count. For example, against MSSA, increasing cationic charge to +2, i.e., K-MSH, caused reduction of 2.03 ± 0.06 Log_10_ CFU/mL whereas +5 charge containing analogue KKK-MSH caused 4.07 ± 0.92 Log_10_ CFU/mL reduction in initial MSSA cell count. Compared to α-MSH statistically significant reduction in CFU count was observed for most active peptide KKK-MSH (p < 0.05). Similar reductions in viability were also observed in the case of MRSA (Fig. 3b). Interestingly, the analogues with the higher charges (+4 and +5) were found to be more active against MRSA compared to analogues with lower charges (+2 and +3). For example, for K-MSH, 1.04 ± 0.49 Log_10_ CFU/mL reduction was observed whereas for KKK-MSH 3.81 ± 0.33 Log_10_ CFU/mL reduction in cell count was observed compared to initial cell count.

Thus, among the designed analogues of α-MSH, KKK-MSH showed maximum staphylocidal efficacy against both MSSA as well as MRSA indicating the strong contribution of enhanced cationicity of the peptides towards their activity.

### α-MSH and the designed peptides were non-toxic towards mammalian cells and enhanced cyclic-AMP levels in mouse melanocytes

**Figure 4:**
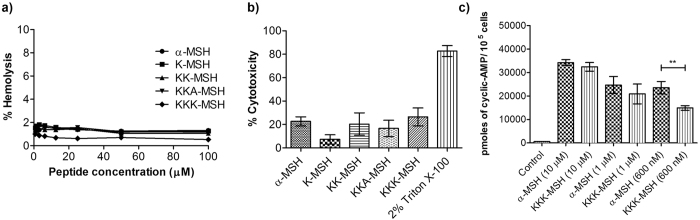
Hemolysis, cytotoxicity and cyclic-AMP accumulation upon α-MSH and designed analogues treatment. In (**a**) concentration-dependent hemolysis induced upon 1 h incubation of mouse RBCs with peptides. In (**b**) % cytotoxicity against 3T3 murine fibroblast cell line at 50 μM peptide concentration. In (**c**) induced accumulation of cyclic-AMP in B16 melanocytes on exposure to different concentrations of α-MSH and KKK-MSH. Each assay was done in triplicate on three different days and each data point represents mean ± S.D.

The non-specific toxicity of peptides towards mammalian cells remains a major obstacle to the development of peptide-based therapeutics^34^. For evaluation of cell selectivity of the peptides, we measured their effect on mouse red blood cells and murine fibroblast cell line. As evident from Fig. 4a, all designed analogues, as well as α-MSH, showed negligible hemolytic activity (<2% hemolysis) even up to concentration of 100 μM.

Further, cytotoxicity studies were carried out on the analogues against fibroblast 3T3 cell line using MTT assay. As seen from the Fig. 4b, all studied peptides including α-MSH resulted in 7.2 ± 3.8% to 26.4 ± 7.6% cytotoxicity at 50 μM concentration which is relatively high as compared to the concentration required for their antibacterial activity. For the experiment, as a positive control we used 2% Triton X-100 which showed 83% cytotoxicity relative to growth control.

Next, we quantified levels of cyclic-AMP in mouse B16 melanocytes induced upon treatment with varying concentrations of α-MSH and most active analogue KKK-MSH. The results showed significant enhancement in cyclic-AMP levels for both α-MSH and KKK-MSH, suggesting that the treatment of B16 cells with these peptides elicits a dose-dependent elevation in cyclic-AMP compared to untreated control (p < 0.05). While the cyclic-AMP levels at higher concentration of the peptide were comparable between α-MSH and KKK-MSH, at lower concentration the latter elicited lower cyclic-AMP levels, suggesting that charge modification also impacts receptor activation (Fig. 4c).

Altogether, negligible hemolysis and little to no cytotoxicity were observed for all peptides against mammalian RBCs and fibroblast cell line indicating their selectivity for the bacterial membrane. Further, similar to α-MSH our designed peptide KKK-MSH was able to elicit comparable or lower levels of cyclic-AMP in mouse melanocytes.

### KKK-MSH is the most active peptide causing membrane depolarization and permeabilization

**Figure 5:**
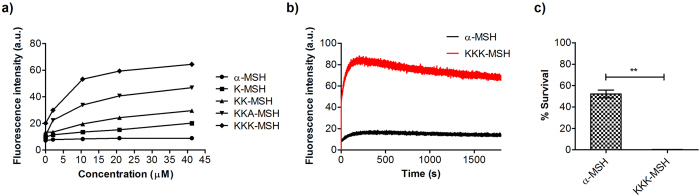
Membrane depolarization mechanism of α-MSH and designed analogues. (**a**) Concentration-dependent membrane depolarization in MSSA ATCC 29213 caused upon treatment with peptides, (**b**) time kinetics of depolarization monitored at fixed peptide concentration (10 μM) and (**c**) % survival of dye loaded cells measured using colony count assay. Mean ± S.D. from two independent experiments is presented here.

We have previously shown that α-MSH and its C-terminal fragments cause membrane depolarization/disruption as part of their killing mechanism against *S. aureus*^23^. Therefore, a comparative study of the efficacies of α-MSH analogues to depolarize the membrane potential of *S. aureus* was carried out using a membrane potential sensitive dye 3,3′-dipropylthiacarbocyanine iodide (DiSC_3_(5)). Upon uptake in live cells, the fluorescence of this probe is quenched and in the presence of a membrane depolarising agent, the dye is released with a considerable increase in its fluorescence, which can be measured fluorometrically. Figure 5a shows a concentration-dependent increase in fluorescence of DiSC_3_(5) on account of membrane depolarization caused by the peptides in *S. aureus*. Under the experimental conditions, α-MSH caused a marginal increase in relative fluorescence intensity (RFI) for the dye, up to the maximum concentration tested.

K-MSH and KK-MSH also exhibited concentration-dependent increase in RFI. For KKA-MSH and KKK-MSH appreciable increase in RFI was observed at 10.47 μM peptide concentration. Thereafter, the RFI stabilized and marginal increase was observed up to the highest concentration tested of 41.2 μM. Representative results from one assay are presented here (Fig. 5a). As the fluorescence changes in this study were measured immediately (~2 min) after addition of peptides to the cells, we further compared time-dependent changes in fluorescent intensity on account of membrane depolarization due to α-MSH and the most active peptide KKK-MSH. The time-dependent kinetics over 30 min showed immediate and enhanced ability of KKK-MSH to alter membrane potential as compared to α-MSH (Fig. 5b). The lethal effect of depolarization on cells was further established by measuring the corresponding viability of DiSC_3_(5) loaded cells at 10 μM peptide concentration (Fig. 5c). This showed a direct correlation between the bactericidal activity and membrane depolarization. Exposure to α-MSH (10 μM) for 5 min killed 47.82 ± 3.61% of *S. aureus* cells whereas same exposure to KKK-MSH killed 99.7 ± 0.05% bacterial cells, confirming the increased potency of KKK-MSH than α-MSH due to its enhanced membrane depolarization capability.

**Figure 6:**
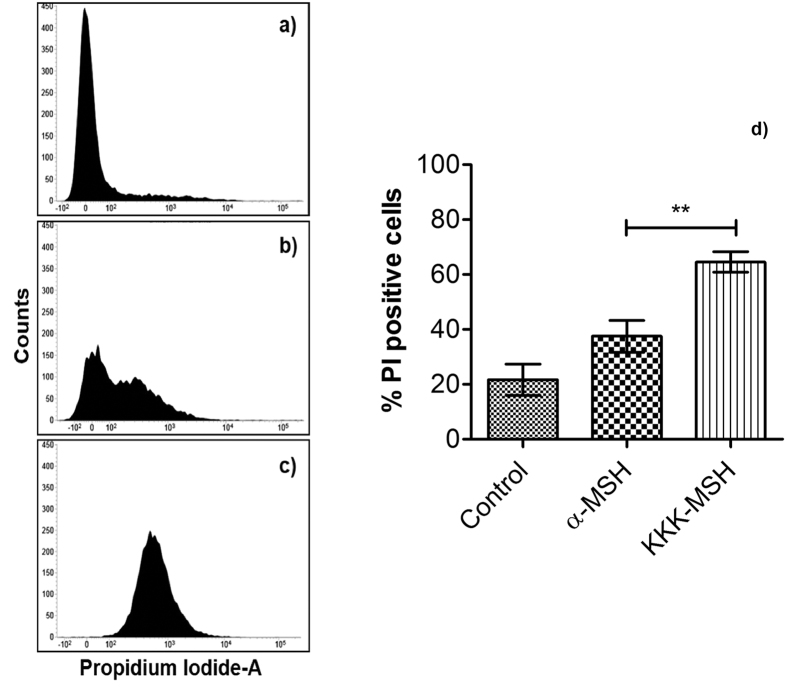
Membrane permeabilization of MSSA ATCC 29213 upon incubation with α-MSH and KKK-MSH. Histograms of cells incubated with PI and treated with different peptides at 10 μM concentration for 1 h. Here, (**a**) untreated cells, (**b**) cells treated with α-MSH, (**c**) cells treated with KKK-MSH, and (**d**) % of PI positive *S. aureus* cells on α-MSH and KKK-MSH treatment. A total of 10,000 cells were acquired for each flow cytometry analysis. Mean ± S.D. from three independent experiments is presented here.

We next compared membrane disruption ability of α-MSH and KKK-MSH against *S. aureus* using a DNA binding fluorescent probe propidium iodide (PI) via flow cytometry. PI, being membrane-impermeant, can enter into the cell only through disrupted membranes and gives fluorescence once it is bound to nucleic acid inside the cells. For this study, cells having >10 a.u. were considered to have taken up PI. Upon peptide treatment, fluorescence peaks were shifted towards higher fluorescence values as can be seen from the histograms (Fig. 6b,c). Specifically, α-MSH treatment for 1 h resulted in 42.9 ± 11.8% cells retaining PI as compared to 67.6 ± 3.4% cells for KKK-MSH. The difference in % of PI positive cells was found to be significant between α-MSH and KKK-MSH (p < 0.05).

Overall, charge-dependent depolarization and permeabilization effects were observed whereby the most cationic peptide KKK-MSH (+5) showed maximum membrane destabilization potential.

### KKK-MSH showed higher lipid binding in artificial bacterial mimic membrane as compared to α-MSH

Further, we evaluated the relative binding constants of α-MSH and KKK-MSH to bacterial mimic SUVs to understand if there are differences between lipid binding affinities of these two peptides. Towards this, we incubated a fixed concentration of peptide (12.5 μM) with increasing concentrations of bacterial mimic SUVs (DMPC:DMPG, 7:3, w/w) and concomitant changes in the intensity of emission maxima for Trp residue were recorded. Correction for light scattering was done by subtracting the corresponding spectra of the SUVs alone. To calculate the binding constant we plotted the fraction of bound peptide (F − F_0_)/(F_max _− F_0_) vs. lipid concentration and fitted the data to a hyperbolic curve using Origin 8 (2015) software. The lipid to peptide ratio was varied from 75.6 to 3.3.

**Figure 7:**
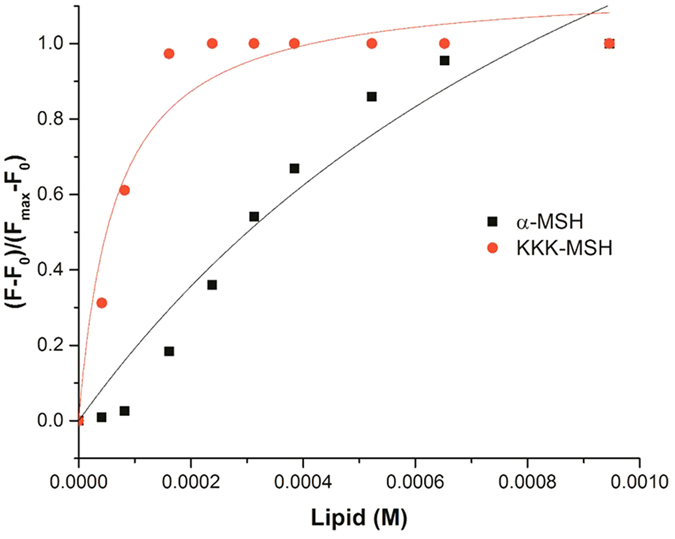
Trp fluorescence studies to estimate binding of α-MSH and KKK-MSH to bacterial mimic SUVs. Increasing concentrations of DMPC:DMPG (7:3, w/w) SUVs in 10 mM TES buffer (pH 7.2) were titrated with fixed peptide concentration (12.5 μM) and the emission spectrum was recorded. The binding curve was obtained by plotting the fraction of bound peptide (F − F_0_)/(F_max_ − F_0_) vs. SUVs concentrations. The data points were fitted to a hyperbolic curve using Origin software. The fluorescence spectrum was recorded at 25 °C by exciting samples at 295 nm and recording emission from 310 nm to 450 nm. A slit width of 3 nm was used for both excitation and emission. Contributions from the buffer and SUVs were appropriately subtracted and volume corrected fluorescence values were used to calculate the fraction of bound peptide.

For α-MSH, the results showed a gradual increase in fraction of bound peptide (measured as Trp fluorescence intensity) on increasing lipid concentration whereas, for KKK-MSH, a steep rise in fluorescence intensity was observed leading to saturation at very low lipid concentration, indicating higher affinity of KKK-MSH towards bacterial mimic SUVs (Fig. 7). Consequently, the corresponding binding constants obtained were 826.44 M^−1^ for α-MSH and 15,402.74 M^−1^ for KKK-MSH. The results thus suggested that the initial interaction of the peptide with membrane may be electrostatic (direct interaction with membrane lipid). Hence, the relative binding data clearly supports more prominent binding to and destabilization of bacterial membranes by KKK-MSH as compared to α-MSH.

### Alteration in bacterial morphology upon exposure to the peptides observed via electron microscopy

**Figure 8:**
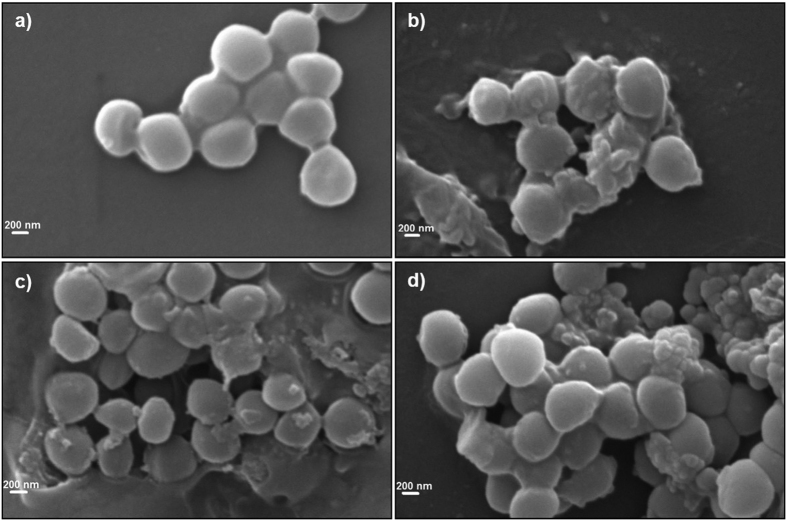
Morphological changes observed after exposure of MSSA ATCC 29213 to α-MSH and KKK-MSH using scanning electron microscopy. (**a**) Untreated control, (**b**) α-MSH (100 μM), (**c**) KKK-MSH (100 μM) and (**d**) Gramicidin D (20 μg/mL).

To visualize the effect of peptide treatment on the morphology of *S. aureus* via scanning electron microscopy, we incubated α-MSH (100 μM), KKK-MSH (100 μM) and gramicidin D (20 μg/mL) with 10^9^ CFU/mL for 2 h. Figure 8a shows untreated control cells having normal round cell morphology with a smooth surface as compared to α-MSH treatment (Fig. 8b) which resulted in severe alterations in membrane shape/architecture and surface protrusions, as observed by us previously^23^.

KKK-MSH treated cells also showed altered morphology with several surface blebs and intracellular material oozing out due to bursting of the cells (Fig. 8c). Upon gramicidin D treatment, cell debris with mini cell formation was observed (Fig. 8d). Similar membrane defects have been shown to occur for gramicidin D in our previous work under identical conditions^23^.

## Discussion

A substantial need towards the discovery of novel antibacterial agents, preferably with multiple targets in microbes, is being felt owing to the current lack of freshly approved systemic antibacterial agents^35^,^36^. A number of host-derived peptides or their mimics with direct bactericidal activity along with other essential cellular targets present their candidature towards optimization as antibacterial leads^3^,^37^,^38^. Our previous studies have shown that the neuropeptide α-MSH and its smaller fragments i.e., α-MSH(6–13) and α-MSH(11–13) have potent activity against *S. aureus*, MRSA and several clinical strains of *S. aureus* in micromolar concentration range^22^,^23^,^24^,^25^,^26^. Recently, in an elegant attempt to modify the activity of α-MSH(6–13), Grieco *et al*. incorporated several unnatural amino acids in the structure of the peptide which promoted α-helical conformation along residues 8–13^21^. They successfully obtained more potent analogues with slight detrimental effects towards human keratinocytes (HaCat cell line) albeit at greater concentrations relative to their effective antibacterial concentrations. Mechanistically, the primary target for α-MSH was found to be membrane disruption, firstly by depolarizing and then permeabilizing the cell membrane of *S. aureus*^23^. In artificial membranes also, we showed preferential interaction of cationic α-MSH with anionic bacterial mimic membranes^26^. This could be thought to be mediated by electrostatic interactions between positively charged α-MSH (+1) and negatively charged bacterial phospholipid membranes. Therefore, to further elucidate the role of cationic charge on α-MSH activity, we designed four novel α-MSH analogues with enhanced cationic charges.

A balance between hydrophobicity and cationic charge has been reported to be crucial for the activity of many membrane active CAMPs^39^,^40^,^41^. In our present work, we observed that upon increasing the charge from +1 to +5, there was no appreciable difference in hydrophobicity of the analogues based on RP-HPLC (C18 column) (Table 1). α-MSH showed characteristic CD spectrum as has been previously reported^26^ in buffer and DMPG SUVs (Fig. 1). Compared to α-MSH, the relatively unchanged hydrophobicity of the designed peptides also got reflected in their secondary structure as mostly similar structural features were observed for all peptides (Fig. 1). For α-MSH it has been shown that the minimum energy conformation is a β-turn which is present in the middle of the sequence spanning the putative message region (residues 6–9)^42^. Since we incorporated modifications in the N-terminal region (residues 1–5), which does not take part in the turn formation, our results indicate preservation of native α-MSH conformation in all the designed peptides in both the tested lipid compositions (DMPC:DMPG, 7:3 and 1:1, w/w) at low and high L:P ratios (see Supplementary Fig. S1). Additionally, all the peptides showed similar conformation in buffer and bacterial mimic SUVs. Similarly, a study by Eiríksdóttir *et al*. demonstrated that a subgroup of cationic cell-penetrating peptides like Tat and Arg_9_ did not exhibit any structural changes and retained their random coil form in the presence of phospholipid bilayers^43^. Although, acquisition of secondary structure in the vicinity of phospholipid bilayers has been reported to be crucial for activity, there are examples where no direct correlation between peptide structure acquisition ability and antibacterial activity was observed. For example, C-terminal amidated aurein 2.3 and its corresponding C-terminal acid peptide both change their conformation from unordered in buffer to α-helical in DMPC and DMPC:DMPG SUVs, however aurein 2.3-COOH remains largely inactive against the tested bacterial strains as compared to aurein 2.3^44^,^45^.

We next measured the insertion depth of the peptides in bacterial and mammalian mimic SUVs using intrinsic Trp residue as a probe. All the designed peptides and α-MSH inserted preferentially into mixed (anionic/zwitterionic) bacterial mimic membranes as compared to zwitterionic mammalian mimic membranes, emphasizing on the role of enhanced electrostatic interactions between negatively charged membranes and cationic peptides (Fig. 2). However, a linear correlation between increased charge and insertion depth in bacterial mimic SUVs was not observed (Table 2). This may result from the relatively unchanged/conserved hydrophobicity of our designed analogues as the core segment hydrophobicity of peptides has been shown to directly influence their insertion into bacterial membrane^27^,^46^. Similar results have been reported for cationic analogues of puroindoline derived Trp-rich antimicrobial peptides where analogues with increased charge did not lead to enhanced blue shifts in bacterial mimic membranes^47^. Interestingly, a progressive increase in staphylocidal potential was observed for the designed peptides upon increasing cationic charge and KKK-MSH being the most charged peptide showed significant improvement in killing efficacy as compared to α-MSH (p < 0.05) (Fig. 3). Similar results were obtained in a study by Lee *et al*. where the modified analogue with increased cationicity, Anal R (net charge +5), showed enhanced killing efficacy against both Gram-positive and Gram-negative bacteria as compared to the parent Rev-NIS (net charge +2) and another analogue Anal S (net charge +2)^48^. Along with the increase in antibacterial potency, there were no detrimental effects of designed peptides on mammalian cells as less than 2% hemolysis was observed at concentrations much higher than the dose required for their antibacterial effect (Fig. 4a). Cytotoxicity study on mouse 3T3 fibroblast cell line also established little to no toxicity of α-MSH and its analogues (Fig. 4b). Thus, our design towards optimization of potency of α-MSH while maintaining cell selectivity was successful. There are numerous reports in literature which show a direct correlation between increased hydrophobicity and toxicity^49^,^50^,^51^. Since we maintained the hydrophobicity in our novel designed peptides, therefore as expected we observed negligible hemolysis and low cytotoxicity. Additionally, as compared to α-MSH, our preliminary results showed KKK-MSH to be able to elicit comparable or lower levels of cyclic-AMP in mouse melanocytes (Fig. 4c). In previous reports^12^,^19^, α-MSH induced elevated cyclic-AMP levels have been shown to elicit antifungal and anti-inflammatory effects in *Candida albicans* and mammalian cells, respectively. Therefore, we speculate that our designed analogue KKK-MSH may also have similar beneficial biological effects.

As we have already shown the primary target of α-MSH to be the bacterial membrane in our earlier studies^23^, we next evaluated and compared mechanism of action of designed peptides on *S. aureus*. Towards this, we first measured alteration in membrane potential of *S. aureus* upon treatment with increasing peptide concentration. A clear charge-dependent correlation of membrane depolarization ability was observed whereby KKK-MSH exhibited maximum extent of membrane depolarization (Fig. 5). Similar results for charge-dependent depolarization ability of membrane active CAMPs have been reported previously^52^. Along with membrane depolarization, KKK-MSH caused membrane permeabilization as evident from PI uptake assay (Fig. 6). Since membrane binding correlates with membrane disruption, therefore, we evaluated relative binding constants of α-MSH and KKK-MSH in bacterial mimic membranes. The binding constant for KKK-MSH was found to be ~18-fold higher than for α-MSH as was determined by steady-state fluorescence (Fig. 7). Thus, KKK-MSH has a higher association constant with lipid, i.e., higher binding to the lipid vesicles. The role of enhanced cationic charge leading to 10-fold improved binding to melanotropic receptors has been shown to promote anti-inflammatory activities for a modified α-MSH analogue AP214 with a hexa lysine tag at N-terminal^53^,^54^. Although in the case of AP214, a receptor-mediated mode of action was involved, still increase in cationicity was among the factors that promoted activity. Our present work further suggests a role for enhanced cationicity in augmenting the interaction of the peptide with the negatively charged bacterial membranes which translates into staphylocidal activity, thus potentiating the role of enhanced electrostatics in promoting bacterial killing. Ultimately, we visualized the effect of α-MSH and KKK-MSH on cell morphology using scanning electron microscopy. The results showed appreciable changes in morphology of *S. aureus* after exposure to the peptides (Fig. 8b,c), thus confirming their membrane disruptive mode of action.

Overall, increasing the cationic charge improved staphylocidal activity and membrane damaging efficacy of the designed α-MSH analogues (maximum efficacy shown by analogue with +5 charge). This may be attributed to the increased electrostatic interactions leading to the better binding of peptides to negatively charged artificial bacterial membranes. This has been further corroborated in intact *S. aureus* cells as enhanced depolarization and membrane disruption was caused by the analogues without compromising mammalian cell membrane integrity. Our overall findings thus suggest an important role of cationicity of peptide towards improving its antimicrobial activity. At present, we are evaluating anti-MRSA biofilm potential of most active peptide KKK-MSH emerging out of this study.

## Materials and Methods

### Materials

Brain heart infusion media was purchased from Himedia Laboratories, India. Propidium iodide (PI), trifluoroethyl alcohol (TFE), 3,3′-dipropylthiadicarbocyanine iodide (DiSC_3_(5)), DMPC (1,2-dimyristoyl-sn-glycero-3-phosphocholine), DMPG (1,2-dimyristoyl-sn-glycero-3-phospho-rac-(1-glycerol) sodium salt), dextrose and Triton X-100 were purchased from Sigma-Aldrich, USA. 2-[tris(hydroxymethyl)-methylamine]-1-ethanesulfonic acid (TES buffer) and 4-(2-hydroxyethyl)piperazine-1-ethanesulfonic acid (HEPES) were purchased from SRL (India). 3-(4,5-dimethylthiazol-2-yl)-2,5-diphenyltetrazolium bromide (MTT), dimethyl sulfoxide (DMSO) and Dulbecco’s modified eagle medium (DMEM) were purchased from Sigma-Aldrich, USA. Fetal bovine serum (FBS) was purchased from Gibco, India.

#### Bacterial strains

Methicillin-sensitive *S. aureus* (MSSA ATCC 29213) and methicillin-resistant *S. aureus* (MRSA ATCC 33591) were used in the present study. The strains were stored at −70 °C in 15% (v/v) glycerol until sub-cultured onto brain heart infusion (BHI, Himedia Laboratories, India) agar plates for further use.

#### Peptides

Gramicidin D, magainin II and α-MSH were purchased from Sigma-Aldrich, USA. The designed peptides (HPLC purity >98%) were custom synthesized from Biochain Incorporated, India. The concentration of the peptides was determined spectrophotometrically (UV-2450, UV-VIS spectrophotometer, Shimadzu) by measuring the absorbance at 280 nm and using theoretical extinction coefficient (ε) 6.65 × 10^3^M^−1^cm^−1^ for both Trp and Tyr.

### Methodology

#### Preparation of SUVs for biophysical studies

Artificial bacterial mimic (DMPC: DMPG, 7:3 and 1:1, w/w) and mammalian mimic (DMPC) SUVs were prepared by probe sonication using a previously defined method with slight modifications^26^. Briefly, the desired amount of lipids in fixed ratio were dissolved in chloroform-methanol mixture in a 100 mL round-bottom flask. The solvent was evaporated under a stream of nitrogen gas to allow the formation of a thin lipid film, which was dried overnight in a dessicator. Next day, the lipid film was rehydrated with desired buffer (10 mM TES buffer for fluorescence spectroscopy and 5 mM sodium phosphate buffer for CD) in a water bath above the phase transition temperature of the lipids. The lipid suspension was swirled for 30 min in a water bath. Further, the suspension was transferred to a 50 mL falcon tube and probe sonicated for 15 min using burst and halt time of 30 s and 10 s, respectively. The titanium debris was removed by centrifugation. The mean radius distribution for SUVs was found to be in range of 70–90 nm as determined by dynamic light scattering (DLS) Xtal spectrosize 300 (Hamburg, Germany).

#### CD spectroscopy

CD spectra were acquired in different environments including 5 mM sodium phosphate buffer, TFE (50% v/v) and bacterial mimic SUVs (DMPC:DMPG, 7:3 and 1:1, w/w) on Applied PhotoPhysics Chirascan (Surrey, United Kingdom) instrument at 37 °C. A step size of 0.2 nm, bandwidth of 1 nm and pathlength of 1 mm was used to scan 190–260 nm (far UV region) wavelength range. CD spectra of peptides were collected and averaged over three scans with different lipid to peptide ratios. Representative spectra expressed as molar ellipticity (deg.cm^2^.dmol^−1^) vs. wavelength after appropriate blank subtraction is presented here.

#### Trp fluorescence emission studies

The Trp fluorescence emission spectra were acquired on Shimadzu RF-5301 PC spectrofluorimeter with the constant temperature maintained at 25 °C. The insertion depth of designed peptides in model artificial membranes was evaluated using the shift in emission maxima and change in emission intensity of Trp fluorescence as described elsewhere^26^. At a fixed lipid to peptide ratio, each peptide was added to either TES buffer or bacterial or mammalian mimic SUVs. The fluorescence was recorded with an excitation wavelength of 295 nm and the emission was collected from 310 to 450 nm with excitation and emission slit width of 3 nm. Lipid to peptide ratio 58:1 was maintained with a peptide concentration of 12.5 μM and lipid concentration of 726 μM.

#### Antimicrobial activity

To determine the antibacterial activity, cells (MSSA ATCC 29213 and MRSA ATCC 33591) from secondary culture mid-logarithmic phase were used. The cells were centrifuged, washed and re-suspended in buffer (5 mM HEPES, 20 mM glucose, pH 7.4). The re-suspended cells were adjusted to OD_600_ of 0.5 spectrophotometrically (~10^8^ CFU/mL). Desired cell densities were then exposed to 10 μM concentration of α-MSH and other analogues and incubated at 37 °C for 2 h. After 2 h incubation, the cells were diluted in buffer and small aliquots were plated on BHI agar plates. The plates were incubated overnight at 37 °C. Survival was determined by counting of the colony forming unit (CFU/mL) on agar plate using the following formula^23^:


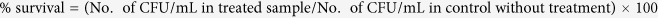


In the present work minimum inhibitory concentration (MIC) using broth micro-dilution technique following Clinical Laboratory Standards Institute (CLSI) guidelines could not be determined since similar to α-MSH the antibacterial activity of designed peptides got abolished in nutrient media used for the experiment^24^,^25^.

#### Hemolytic activity

A protocol as described previously was used to measure hemolytic activity with slight modifications^55^. Briefly, fresh blood from mice was collected on the day of the experiment. The blood was centrifuged at 1,500 rpm for 10 min to remove the plasma and buffy coat. The RBCs pellet was re-suspended in phosphate buffer saline (PBS: 35 mM sodium phosphate, 150 mM NaCl, pH 7.4) to 4% v/v. Serial dilutions of peptides in buffer were prepared in a 96-well plate (100 μL). To this 100 μL of RBCs suspension (4% v/v) was added and incubated at 37 °C for 1 h. After 1 h, the plates were centrifuged at 1,500 rpm for 10 min and 20 μL of the supernatant was added to 80 μL of PBS in a fresh 96-well plate. The hemoglobin release was determined by measuring absorbance at 414 nm in an ELISA plate reader (Molecular Devices, Sunnyvale, CA, USA). For 100% and 0% hemolysis, 0.1% Triton X-100 (v/v) and buffer were used, respectively. % hemolysis was calculated as [(OD_414_ of sample-OD_414_ of PBS)/(OD_414_ of positive control-OD_414_ of PBS)] × 100. The experiment was performed under CPCSEA guidelines [Committee for the Purpose of Control and Supervision of Experiments on Animals] and Institutional Animal Ethics Committee (IAEC-02/2014) of JNU, New Delhi, India.

#### Measuring cytotoxicity by MTT assay

The cytotoxicity of α-MSH and analogues was tested against 3T3 murine fibroblast cell line using a protocol as described previously^25^. The cell line was propagated at 37 °C in 5% CO_2_ to over 75% confluence in DMEM supplemented with 10% fetal bovine serum (FBS) and antibiotics and transferred (after treatment with trypsin) to each well of a 24-well plate to a final count of 0.2 × 10^5^ cells/well for 24 h. After 24 h, media was aspirated and desired concentration of peptides (50 μM) dissolved in 25% (v/v) DMEM in PBS was added to each well (in triplicate). Triplicate wells without treatment were set as control and wells with 2% Triton X-100 were used as positive control. The plate was kept for 2 h, at 37 °C in 5% CO_2_ incubator. After 2 h, media was removed and the cells were washed once with 1 mL PBS. In dark, 1 mL MTT (working conc. 0.1 mg/mL) was added to each well and incubated for 2 h at 37 °C. After 2 h, the formazan crystals formed were dissolved in 200 μL of DMSO and kept for 5 min in the incubator. 150 μL of this mixture was transferred to a fresh 96-well plate and absorbance was measured spectrophotometrically at 570 nm. % cytotoxicity was calculated as [(OD_570_ of control-OD_570_ of sample)/(OD_570_ of control)] × 100. The assay was done in triplicate on three different days.

#### Cyclic-AMP accumulation analysis

The intracellular cyclic-AMP analysis was performed according to a method described elsewhere^56^, with slight modifications. Briefly, B16 mouse melanoma cells stably expressing human melanocortin receptor MC1R were grown to confluence in DMEM medium (Sigma-Aldrich, USA) containing 10% fetal calf serum (FCS) and 1x anti-anti (Gibco). The cells were seeded in 12-well plates 48 h before assay and grown to 10^5^ cells/well. For the assay, the medium was removed and cells were washed with 1 mL of media. Then 1 mL DMEM media (without FCS) containing 100 μM 3-isobutyl-1-methylxanthine (IBMX) was added to each well and incubated for 30 min at 37 °C. The media was then discarded and various concentrations of α-MSH and KKK-MSH viz., 600 nM, 1 μM and 10 μM, in 1 mL fresh 25% DMEM media (without FCS) in PBS containing 100 μM IBMX were added to the appropriate wells and incubated for 10 min at 37 °C, 5% CO_2_. After 10 min the reaction was stopped by aspirating the media and washing the wells with 1 mL PBS. 200 μL 0.1 M hydrochloric acid was then added to each well and the wells were scrapped. The lysates were centrifuged at 600 rcf for 5 min and the supernatants were collected and diluted 1000 times in 0.1 M HCl and used for analysis of the total cyclic-AMP content using Enzyme Immunoassay Kit (Sigma-Aldrich, USA, CA200) according to the manufacturer’s instructions.

#### Membrane depolarization assay

The cytoplasmic membrane depolarization was measured using membrane potential sensitive cyanine dye DiSC_3_(5)^57^. Briefly, mid-logarithmic phase MSSA ATCC 29213 cells were washed and re-suspended in buffer (5 mM HEPES, 20 mM glucose, pH 7.2) to an OD_600_ of 0.05 which corresponds to ~10^7^ CFU/mL. Cells were divided into aliquots and incubated with 2 μM of DiSC_3_(5) for 1 h to get steady baseline fluorescence intensity. The dye-loaded cells were treated with increasing peptide concentrations (2.1 μM, 10.47 μM, 20.83 μM and 41.23 μM). Fluorescence was monitored in a Shimadzu RF-5301 PC spectrofluorimeter at an excitation wavelength of 622 nm and an emission wavelength of 670 nm. The corresponding killing was measured by plating the aliquots onto BHI agar plate to obtain viable bacterial counts.

#### Membrane permeabilization assay

The propidium iodide (PI) uptake assay was performed to assess membrane permeabilization by flow cytometry according to a method described elsewhere^58^. MSSA ATCC 29213 cells were grown in BHI broth to mid-logarithmic phase and harvested by centrifugation at 4,000 rpm for 10 min. The cell pellet was washed once with PBS (10 mM sodium phosphate, 150 mM NaCl, pH 7.4) and was adjusted to 10^5^ CFU/mL in PBS. The cells were incubated with PI (1.3 μg/mL) at 37 °C for 20 min in the dark. After incubating the cells with PI, cells were exposed to peptides (10 μM) at 37 °C. PI fluorescence was measured after 1 h incubation with excitation and emission wavelength of 544 nm and 620 nm, respectively, using Becton Dickinson (BD) FACS verse, San Jose, CA. The cells having more than 10 fluorescence unit (arbitrary) were considered to be stained with PI.

#### Lipid binding

Lipid binding was measured by titrating SUVs (DMPC:DMPG, 7:3, w/w) into 12.5 μM of the peptide by varying lipid to peptide ratio from 75.6 to 3.3. After each addition of lipid, emission spectrum was recorded between 310 to 450 nm using excitation at 295 nm with excitation and emission slit width of 3 nm. Blank spectra were recorded for different SUVs concentrations and subtracted to account for fluorescence scattering in emission maxima measurements^59^. To calculate the binding constants we plotted the fraction of bound peptide (F − F_0_)/(F_max_ − F_0_) vs. lipid concentration and fitted the data to a hyperbolic curve for the least square fit algorithm (one site binding model) using Origin 8 (2015) software. Here F is fluorescence intensity of peptide at various lipid concentrations, F_0_ is the intensity of peptide in buffer and F_max_ is the intensity at maximum lipid concentration tested.

#### Scanning electron microscopy

This study was performed as previously reported^23^. In brief, MSSA ATCC 29213 cells were grown to mid-logarithmic phase, harvested by centrifugation at 4,000 rpm for 10 min and re-suspended in PBS (pH 7.4) to OD_600_ of 1.0 (~10^9^ CFU/mL). In order to ensure lethal activity of peptides, 100 μM concentration was incubated with 10^9^ CFU/mL in PBS (pH 7.4) for 2 h. As a control, standard antibiotic gramicidin D (20 μg/mL) was added to same bacterial cell density for 2 h. After incubation, cells were pelleted down at 6,000 rpm for 10 min and washed with PBS (pH 7.4) thrice. Then the cells were fixed with 2.5% glutaraldehyde in the same buffer at 4 °C overnight. On fixation, cells were washed thrice with PBS (pH 7.4) and series of graded ethanol solutions (30% to 100%) were used for dehydration. Finally cells were dried in a vacuum dessicator and the specimens were coated with 20 nm gold particles using an automatic sputter coater (Polaron OM-SC7640). Then samples were viewed via scanning electron microscopy (EVO 40; Carl Zeiss, Germany).

#### Statistical analysis

Statistical significance was calculated using GraphPad prism 5 software by applying the one-way ANOVA and Bonferroni’s post hoc test to determine the significance of the experiment among the groups.

## Additional Information

**How to cite this article**: Singh, J. *et al*. Enhanced Cationic Charge is a Key Factor in Promoting Staphylocidal Activity of α-Melanocyte Stimulating Hormone via Selective Lipid Affinity. *Sci. Rep.* **6**, 31492; doi: 10.1038/srep31492 (2016).

## Supplementary Material

Supplementary Information

## Figures and Tables

**Table 1:** Name, sequence, charge and % hydrophobicity of α-MSH and designed analogues.

Name	Sequence	Charge (Q)	% H*
α-MSH	Ac-Ser^1^-Tyr^2^-Ser^3^-Met^4^-Glu^5^-His^6^-Phe^7^-Arg^8^-Trp^9^-Gly^10^-Lys^11^-Pro^12^-Val^13^-NH_2_	+1	43.6
K-MSH	Ac-Lys^1^-Tyr^2^-Ser^3^-Met^4^-Glu^5^-His^6^-Phe^7^-Arg^8^-Trp^9^-Gly^10^-Lys^11^-Pro^12^-Val^13^-NH_2_	+2	43.0
KK-MSH	Ac-Lys^1^-Tyr^2^-Lys^3^-Met^4^-Glu^5^-His^6^-Phe^7^-Arg^8^-Trp^9^-Gly^10^-Lys^11^-Pro^12^-Val^13^-NH_2_	+3	41.5
KKA-MSH	Ac-Lys^1^-Tyr^2^-Lys^3^-Met^4^-Ala^5^-His^6^-Phe^7^-Arg^8^-Trp^9^-Gly^10^-Lys^11^-Pro^12^-Val^13^-NH_2_	+4	41.7
KKK-MSH	Ac-Lys^1^-Tyr^2^-Lys^3^-Met^4^-Lys^5^-His^6^-Phe^7^-Arg^8^-Trp^9^-Gly^10^-Lys^11^-Pro^12^-Val^13^-NH_2_	+5	39.9

^*^% hydrophobicity based on % of acetonitrile at RP-HPLC elution of α-MSH and its designed analogues.

**Table 2:** Relative blue shifts in emission maxima of Trp residue of α-MSH and its analogues in different environments.

Name	Blue shift (nm)		
	DMPC	DMPC:DMPG (7:3, w/w)	DMPC:DMPG (1:1, w/w)
α-MSH	2	10	11
K-MSH	3	14	13
KK-MSH	1	14	14
KKA-MSH	1	13	12
KKK-MSH	0	11	12
