# Supplementary material for: Enhanced Cationic Charge is a Key Factor in Promoting Staphylocidal Activity of α-Melanocyte Stimulating Hormone via Selective Lipid Affinity
Source: Sci Rep. 2016 Aug 16;6:31492. doi: 10.1038/srep31492 (PMC4985751; doi:10.1038/srep31492)
Supplement: Supplementary Information [file srep31492-s1.pdf]

Online Supplementary Information

Enhanced Cationic Charge is a Key Factor in Promoting Staphylocidal Activity of α-Melanocyte Stimulating Hormone via Selective Lipid Affinity

Jyotsna Singh[1], Seema Joshi[1], Sana Mumtaz[1], Nancy Maurya[2], Ilora Ghosh[2], Shivangi Khanna[3], Vivek T. Natarajan[3] and Kasturi Mukhopadhyay[1,*]

[1]Antimicrobial Research Laboratory, School of Environmental Sciences, Jawaharlal Nehru University, New Delhi-110067, India.

[2]Biochemistry and Environmental Toxicology Laboratory, School of Environmental Sciences, Jawaharlal Nehru University, New Delhi-110067, India.

[3]CSIR-Institute of Genomics and Integrative Biology, Mathura Road, New Delhi-110020, India.

*Email: kasturi26@hotmail.com or kasturim@mail.jnu.ac.in

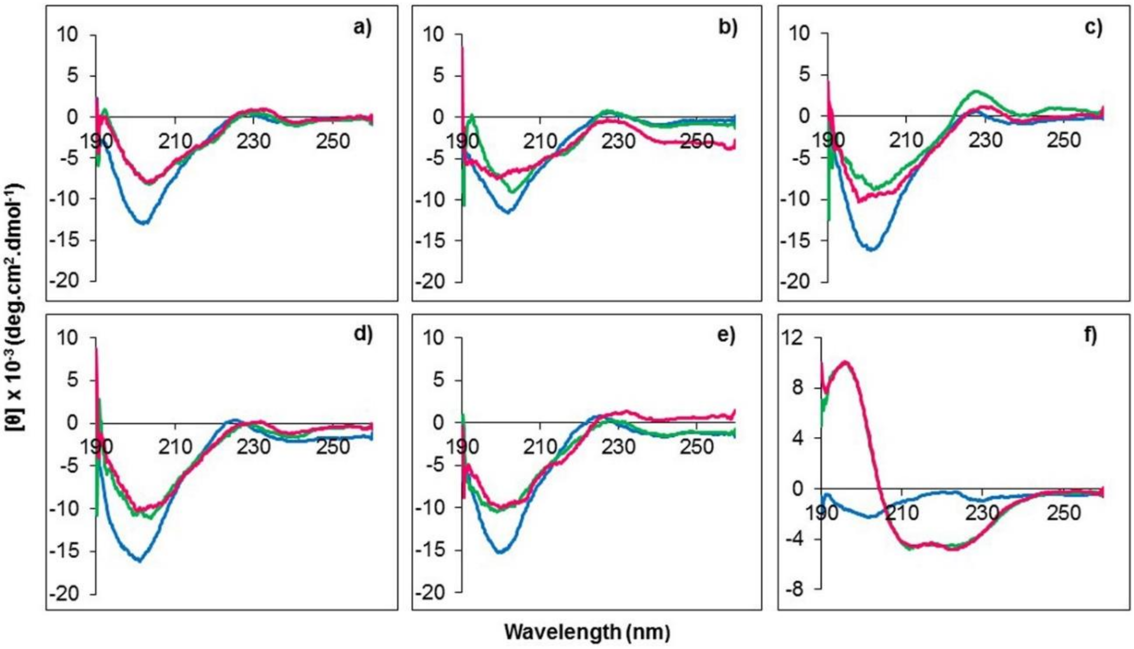

Supplementary Fig. S1: CD spectra of α-MSH and designed analogues. (a) α-MSH, (b) K-MSH, (c) KK-MSH, (d) KKA-MSH, (e) KKK-MSH and (f) Magainin II in the presence of buffer (blue), DMPC:DMPG (7:3, w/w) SUVs (green) and DMPC:DMPG (1:1, w/w) SUVs (pink). Peptide and lipid concentrations were 25 μM and 2,500 μM, respectively (L:P = 100:1). The mean residue ellipticity was plotted against wavelength. Each plot represents average of three scans.
